# High Throughput Centrifugal Electrospinning of Polyacrylonitrile Nanofibers for Carbon Fiber Nonwovens

**DOI:** 10.3390/polym13081313

**Published:** 2021-04-16

**Authors:** Andreas Hoffmann, Alexander J. C. Kuehne

**Affiliations:** 1 Institute of Organic and Macromolecular Chemistry, Ulm University, Albert-Einstein-Allee 11, 89081 Ulm, Germany; andreas.hoffmann@uni-ulm.de; 2 DWI—Leibniz Institute for Interactive Materials, Forckenbeckstraße 50, 52076 Aachen, Germany

**Keywords:** rotational spinning, electrospinning, PAN nanofibers, nonwovens

## Abstract

Carbon nanofiber nonwovens are promising materials for electrode or filtration applications; however, their utilization is obviated by a lack of high throughput production methods. In this study, we utilize a highly effective high-throughput method for the fabrication of polyacrylonitrile (PAN) nanofibers as a nonwoven on a dedicated substrate. The method employs rotational-, air pressure- and electrostatic forces to produce fibers from the inner edge of a rotating bell towards a flat collector. We investigate the impact of all above-mentioned forces on the fiber diameter, morphology, and bundling of the carbon-precursor PAN fibers. The interplay of radial forces with collector-facing forces has an influence on the uniformity of fiber deposition. Finally, the obtained PAN nanofibers are converted to carbon nonwovens by thermal treatment.

## 1. Introduction

Carbon nanofiber nonwovens (CFNs) are a promising class of materials with versatile properties for the wider field of energy applications. CFNs exhibit high conductivity [1], (electro-)chemical resistance [2], high surface area [3], flexibility [4], tunable pore-size and-shape [5,6]. These properties render CFNs applicable in filter media [7,8], wearable electronics [4], electromagnetic shielding [9,10], catalyst supports [11,12], and electrode materials [13].

Unfortunately, due to the limitations in throughput for the production of nanofiber nonwovens, their commercialization has been obviated. To produce carbon precursor fibers, materials like rayon, pitch, or polyacrylonitrile (PAN) are subjected to a spinning process [14]. Whereas spinning of PAN-fibers with diameters on the micrometer scale can easily be facilitated by (dry jet) wet-spinning in a productive and scalable fashion [15,16], pushing the diameter into the sub-micrometer regime requires more sophisticated methods of fiber spinning. PAN-nanofibers are often produced by electrospinning [17]. Here, an electric field between the nozzle and a conductive substrate leads to extension of a Taylor cone and jet formation from the nozzle, which becomes instable and starts whipping, leading to dry and disordered deposition of a nonwoven with fiber diameters in the sub-micrometer range. Conversely, the production rate of electrospinning is limited to about 0.5 g·h^−1^ per nozzle [18]. Up-scaling by parallelization, where the number of nozzles is increased overcomes this limitation; however, this requires precise design of a homogeneous electric field, which complicates the electrospinning setup [19]. To overcome these problems, free surface electrospinning devices have been developed [20]. Here, several spinning jets can be drawn from a reservoir of PAN-dope solution, considerably increasing the throughput in comparison to nozzle-based electrospinning [21]. Recent approaches employ rotational spinning [22,23] or combine the electric field with rotational spinning [24,25,26]. However, the emerging fibers are collected between rods, placed around the spinneret. This results in fiber meshes with a preferred fiber direction and renders continuous production and deposition on flat substrates challenging. The addition of directional air pressure can increase the throughput and therefore the productivity of nanofiber fabrication [27]. Here, the dope solution is fed onto the inner cone of a rotating bell. Centrifugal force constrains the dope solution to flow towards the edge of the bell, where Taylor cones develop, producing fiber jets that are guided to the collector by an electric field as well as by coaxially flowing ‘forming air’ (see Figure S1). The combination of all three forces enables high throughput nanofiber spinning and deposition on a planar collector. Throughputs as high as 15.6 kg·h^−1^m^−2^ have been reported for this nanofiber spinning technique, exceeding all other methods of nanofiber spinning. A small set of water soluble polymers have been spun into nanofibers using centrifugal electrospinning, among them polylactic acid, polyethylene oxide and spider silk [27,28]. While these results are very promising, unfortunately, the influence of the different forces on centrifugal electrospinning of PAN from high boiling solvents is not fully understood, leading to an ill-defined parameter space, which can entail wet fiber deposition, nonwovens with large variation in thickness, bundling of fibers, and polydisperse fiber diameters. These problems have prevented the application of centrifugal electrospinning in the production of carbon fiber nonwovens to date.

Here, we explore the parameter space of this centrifugal electrospinning techniques using DMSO-based PAN solutions. We establish how to control fiber diameter, prevent fiber bundling and obtain densely meshed and homogeneous nonwovens on a flat aluminium foil collector. The parameters will help to transition this technique into commercial applications, with potential for continuous fabrication of carbon nanofiber nonwovens.

## 2. Results and Discussion

### 2.1. Influence of All Processing Forces on the Fiber Diameter

**Figure 1:**
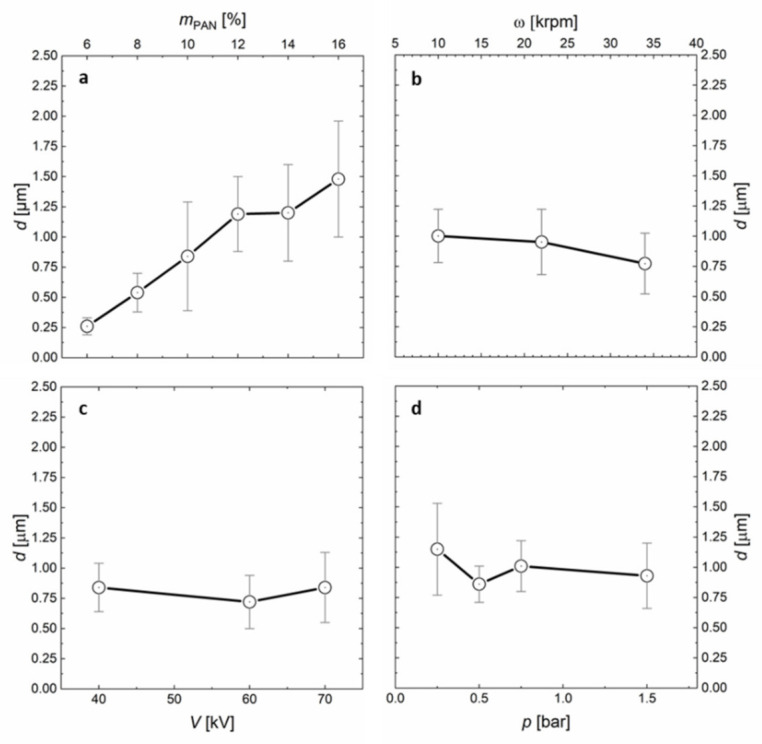
Influence of processing parameters on the fiber diameter. (**a**) PAN-concentration *m*_PAN_ (as a proxy for viscosity), (**b**) rotational speed ω, (**c**) electric field strength *V* and (**d**) air-flow *p* plotted versus the average fiber diameter *d* of the resulting PAN-nonwoven (see Experimental Section in the Supplementary Information). The relative humidity is kept constant at 30% and *I* = 150 μA in (**a**–**d**), *m*_PAN_ = 14 wt% (except in **a**), ω = 30 krpm (except in **b**), *V* = 70 kV (except in **c**), *p* = 0.75 bar (except in **d**). In (**a**) the ω had to be adjusted (increased from 16–26 krpm for increasing *m*_PAN_) to be able to collect fibers at all.

The average fiber diameter *d* of carbon fibers determines the surface-to-volume ratio and therefore has tremendous impact on the performance of the nonwoven in its designated application. In dry spinning processes, the fiber diameter is determined by an interplay of drawing forces on the one hand and the polymer viscosity on the other hand. By contrast, in centrifugal electrospinning we find that voltage (*V*), forming air pressure (*p*), and rotational speed (*ω*) of the rotating bell have only negligible impact on the fiber diameter (see Figure 1). Variation of *ω* between 10–35 krpm decreases *d* from 980–670 nm. Increasing *p* from 0.25 to 1.5 bar reduces *d* from 1150 to 930 nm. Surprisingly, the electric field strength *V* does not seem to have an influence on the fiber diameter at all, while it must play an important role in initial fiber formation, as no fibers are observed in the absence of an electric field (see Figure S2). We hypothesize that the electric field has insignificant effect on *d*, because the other forces compensate for changes in extensional drawing forces. Moreover, the voltage is influenced by humidity, collector size and shape, and is therefore prone to inconsistencies. 

**Figure 2:**
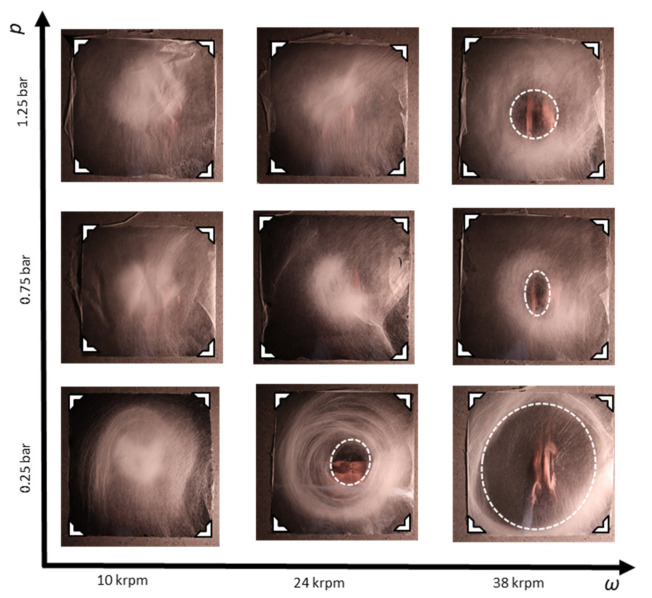
Interplay of radial centrifugal forces and coaxially directed forming airflow on the fiber deposition pattern. The aluminium collector measures 45 × 45 cm, the corners are indicated by white triangles.

By contrast, only the polymer viscosity, adjusted by the mass fraction *m*_PAN_ of polyacrylonitrile in the dope solution, has linear influence on the fiber diameter (cf. Figure 1a with Figure 1b–d). The smallest, spinnable fiber diameter is restricted by the minimum polymer entanglement concentration, which is a known effect also for other nanofiber spinning processes [29,30]. In our case, *m*_PAN_ = 6 wt% in a mixture of 4:1 DMSO:acetone, represents this lower processing limit (see Figure S3). The upper processing boundary of *m*_PAN_ = 16 wt% is set by the highly viscous dope solution, which starts clogging the rotating bell and the connected pipes. Tuning of the PAN dope viscosity between these boundaries enables adjustment of the average fiber diameter between *d* = 260 nm and *d* = 1.48 µm. While the electric field and the airflow are pointing towards the substrate, centrifugal forces are radially pointing away from the collector. The coaxial forces and the centrifugal force sum up to a combined fiber drawing force vector (see Figure S1). The direction of this vector influences the location of the deposited fiber on the collector. Higher rotating speeds lead to fiber deposition on a larger area, up to a point where no fibers are deposited directly underneath the rotating bell, leading to a donut-shaped deposition. Increasing the flow of forming air leads to deposition focused directly underneath the rotating bell. These two extremes are problematic. Donut-shaped deposition leads to circular co-alignment of the individual fibers and less contact points, hampering the desired mechanical properties. Focused deposition entails a shorter flight time of the fibers and therefore wet deposition, which deteriorates the fiber morphology and shape. Dope solutions with *m*_PAN_ = 14 wt% represent an ideal concentration that entails fast drying fibers with acceptable throughput. The optimal conditions for homogeneous fiber deposition for *m*_PAN_ = 14 wt% are found at ω = 28 krpm and *p* = 0.75 bar (see Figure 2).

### 2.2. Fiber Homogeneity within the Nonwoven

**Figure 3:**
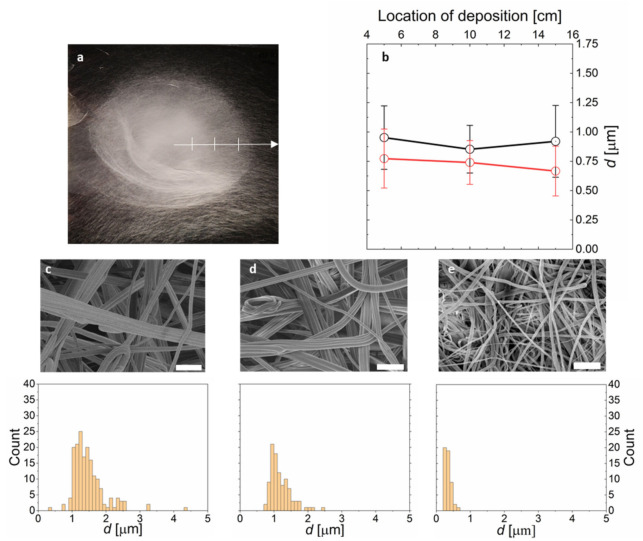
Influence of fiber location on the fiber diameter and distribution of fiber diameters for various nonwovens. (**a**) Photograph of a deposited nonwoven on aluminium foil. The crosspoints along the arrow indicate the points of sampling for (**b**) from the center to the periphery of the nonwoven. (**b**) Fiber diameter plotted versus the distance from the center point for samples indicated in (**a**) (red: 34 krpm; black: 22 krpm). (**c**–**e**) SEM images and fiber diameter histograms of PAN nanofiber nonwovens, spun from (**c**) *m*_PAN_ = 16 wt%, (**d**) 12 wt%, and (**e**) 8 wt%. The scalebars in (**c**–**e**) represent 10 µm.

A narrow and uniform distribution of fiber diameters across the nonwoven is important for many applications of CFN, as variation in *d* affects conductivity, catalytic activity, mechanical strength, and flexibility of the fibers. Centrifugal electrospinning enables large area deposition with widths of about 50 cm from a single spinning source. One might suspect that fibers deposited on the outer edge might have a different diameter than those deposited more towards the center of the deposited nonwoven. Therefore, we determine *d* at three different points; from the center radially towards the edges of the deposited nonwoven (see Figure 3a). *d* is determined by averaging over at least 50 fibers measured in scanning electron micrographs (SEM). We find that *d* does not depend on the location of the fiber, showing that on average, all emerging fibers are exposed to the same amount of extensional forces (see Figure 3).

The distribution of fiber diameters is narrower for low viscosity PAN solutions, with standard deviation of *σ* = 26% for *m*_PAN_ = 6 wt% (*d* = 0.26 ± 0.07 µm) versus *σ* = 32% for a *m*_PAN_ = 16 wt% (*d* = 1.48 ± 0.48 µm). 

**Figure 4:**
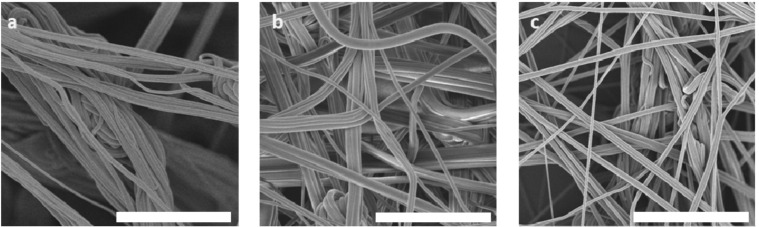
Effect of applied current on the fiber morphology. (**a**) 75 µA, (**b**) 150 µA, and (**c**) 300 µA. The scale bars in (**a**–**c**) represent 30 µm.

Furthermore, for PAN nanofibers produced from more viscous solutions, we observe aggregation of fibers into bundles (see Figure 3 and Figure 4). This bundling might be surprising at first sight, given the fact that voltages as high as 70 kV are employed, which should lead to Coulombic repulsion of the charged fibers during spinning. However, the amount of deposited surface charge per fiber is controlled by the electric current and the surface-to-volume ratio of the fiber. We observe that increasing the current from 75 to 300 µA (instrumental maximum) during spinning leads to fiber separation and deposition of individual fibers in the nonwoven (Figure 4). Similarly, increasing the fiber surface by reducing the fiber diameter (by reducing *m*_PAN_) leads to deposition of less bundled fibers (see Figure S4). From these observations, we conclude that bundling might be the result of van der Waals attraction between fibers, which can be overcome by Coulomb repulsion via deposition of sufficient surface charges on the fibers for reduced fiber diameters and increased spinning currents.

### 2.3. Thermal Treatment for Carbon Nanofiber Nonwovens

**Figure 5:**
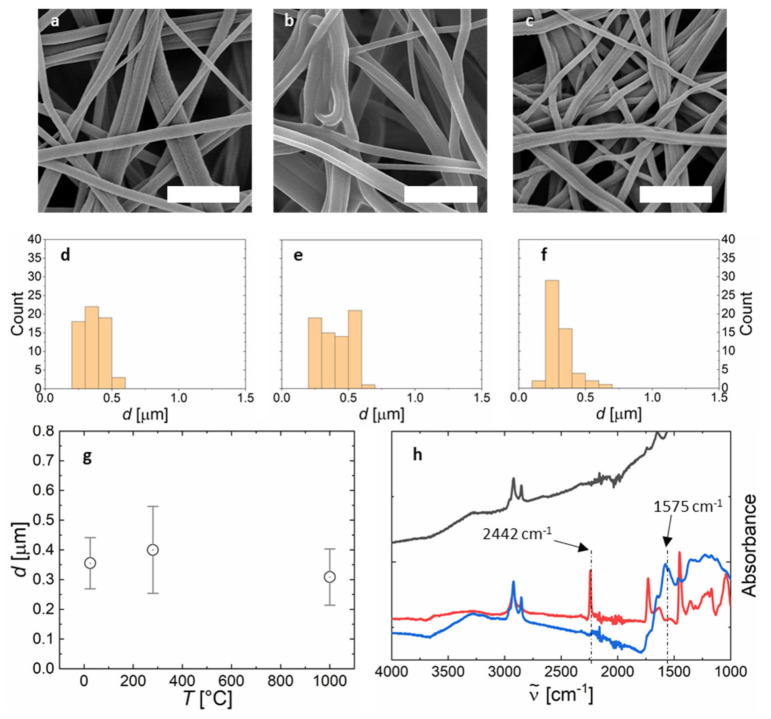
Carbonization of nonwovens produced by centrifugal electrospinning. SEM-images of (**a**) pristine, (**b**) stabilized, and (**c**) carbonized PAN nonwoven. Fiber diameter histograms of (**d**) pristine, (**e**) stabilized, and (**f**) carbonized PAN nonwoven. (**g**) Changes in the average fiber diameter as a function of the thermal treatment temperature. (**h**) IR-Absorbance of pristine (red), stabilized (blue), and carbonized (black) PAN nonwovens. The scalebars in (**a**–**c**) represent 3 µm.

The obtained PAN-nonwovens are free-standing and can be separated from the aluminium substrate, if desired (see Figure 5a). These free-standing PAN nanofiber nonwovens are converted into carbon fibers in a two-step thermal process. First, stabilization is conducted in ambient atmosphere at 280 °C. During stabilization, the PAN chains are transformed into ribbons with slightly enhanced chain stiffness and an increased glass transition temperature enabling further thermal processing. The fiber diameter *d* increases slightly during stabilization due to the chemical conversion within each fiber (see Figure 5b,g). Secondly, carbonization is performed under nitrogen at 1300 °C. Carbonization is accompanied by mass loss resulting in a reduced average fiber diameter from *d* = 355 to 309 nm (see Figure 5c,g). To provide evidence of the chemical conversion, we performed IR-spectroscopy, differential scanning calorimetry (DSC), and thermogravimetric analysis (TGA). The nitrile peak of PAN at 2442 cm^−1^ disappears after stabilization and an imine peak at 1575 cm^−1^ appears, representing the conversion of the nitrile groups into the polymer ribbon structures [31]. The DSC trace of the stabilized nonwoven shows absence of the stabilization exotherm at 300 °C (see Figure S5b). The IR-spectrum changes after carbonization to the typical broad and featureless IR-spectrum of fully converted carbon (see Figure 5h) [32]. TGA shows no decomposition up to 900 °C as is typical for carbon fibers (see Figure S5a).

## 3. Conclusions

We have presented a high throughput method for spinning of PAN nanofiber nonwovens. We have established the parameter space for obtaining separated and sub-micrometer nanofibers as part of a nonwoven fleece. These nonwovens can be converted into carbon by typical thermal treatment, rendering these systems potentially applicable for a large variety of applications, where large surface-to-volume ratios matter, for example, as supercapacitor electrodes, catalyst supports for photo-electrochemistry and filters and adsorbents.

## Data Availability

Data is contained within the article and Supplementary Materials.

## Supplementary Materials

The following are available online at https://www.mdpi.com/article/10.3390/polym13081313/s1, Experimental section, Figure S1: Scheme of the centrifugal electrospinning setup, Figure S2: Variation of voltage from 0 kV to 70 kV, Figure S3: Influence of fiber diameter on fiber morphology, Figure S4: Deposition of droplets, when spinning from *m*_PAN_ = 6 wt%, Figure S5: Thermal analysis of PAN-nonwoven.

## References

[1] Opitz M., Go D., Lott P., Müller S., Stollenwerk J., Kuehne A.J.C., Roling B. (2017). On the interplay of morphology and electronic conductivity of rotationally spun carbon fiber mats. J. Appl. Phys..

[2] Cousin P., Hassan M., Vijay P.V., Robert M., Benmokrane B. (2019). Chemical resistance of carbon, basalt, and glass fibers used in FRP reinforcing bars. J. Compos. Mater..

[3] Kim W.J., Yoon J.Y., Ko T.H., Kim B.S., Chung Y.S. (2018). Fabrication of electrochemical double-layer capacitor electrode using an activated carbon fiber network. Carbon Lett..

[4] Wang C., Xia K., Wang H., Liang X., Yin Z., Zhang Y. (2019). Advanced Carbon for Flexible and Wearable Electronics. Adv. Mater..

[5] Sevilla M., Ferrero G.A., Fuertes A.B. (2017). Beyond KOH activation for the synthesis of superactivated carbons from hydrochar. Carbon N. Y..

[6] Wang J., Kaskel S. (2012). KOH activation of carbon-based materials for energy storage. J. Mater. Chem..

[7] Kim H.J., Han B., Woo C.G., Kim Y.J., Lim G.T., Shin W.G. (2017). Air Cleaning Performance of a Novel Electrostatic Air Purifier Using an Activated Carbon Fiber Filter for Passenger Cars. IEEE Trans. Ind. Appl..

[8] Qin X., Subianto S. (2016). Electrospun Nanofibers for Filtration Applications.

[9] Hong X., Chung D.D.L. (2017). Carbon nanofiber mats for electromagnetic interference shielding. Carbon N. Y..

[10] Yang S., Lozano K., Lomeli A., Foltz H.D., Jones R. (2005). Electromagnetic interference shielding effectiveness of carbon nanofiber/LCP composites. Compos. Part A Appl. Sci. Manuf..

[11] Zhao Y., Lai Q., Zhu J., Zhong J., Tang Z., Luo Y., Liang Y. (2018). Controllable Construction of Core–Shell Polymer@Zeolitic Imidazolate Frameworks Fiber Derived Heteroatom-Doped Carbon Nanofiber Network for Efficient Oxygen Electrocatalysis. Small.

[12] Serp P., Corrias M., Kalck P. (2003). Carbon nanotubes and nanofibers in catalysis. Appl. Catal. A Gen..

[13] Zhang B., Kang F., Tarascon J.M., Kim J.K. (2016). Recent advances in electrospun carbon nanofibers and their application in electrochemical energy storage. Prog. Mater. Sci..

[14] Fitzer E. (1989). Pan-based carbon fibers-present state and trend of the technology from the viewpoint of possibilities and limits to influence and to control the fiber properties by the process parameters. Carbon N. Y..

[15] Nunna S., Blanchard P., Buckmaster D., Davis S., Naebe M. (2019). Development of a cost model for the production of carbon fibres. Heliyon.

[16] Frank E., Steudle L.M., Ingildeev D., Spörl J.M., Buchmeiser M.R. (2014). Carbon fibers: Precursor systems, processing, structure, and properties. Angew. Chem. Int. Ed..

[17] Zhang L., Aboagye A., Kelkar A., Lai C., Fong H. (2014). A review: Carbon nanofibers from electrospun polyacrylonitrile and their applications. J. Mater. Sci..

[18] Li H., Yang W. (2016). Electrospinning Technology in Non-Woven Fabric Manufacturing. Non-woven Fabrics.

[19] Zheng G., Jiang J., Chen D., Liu J., Liu Y., Zheng J., Wang X., Li W. (2019). Multinozzle high efficiency electrospinning with the constraint of sheath gas. J. Appl. Polym. Sci..

[20] Partheniadis I., Nikolakakis I., Laidmäe I., Heinämäki J. (2020). A mini-review: Needleless electrospinning of nanofibers for pharmaceutical and biomedical applications. Processes.

[21] Yalcinkaya F. (2019). Preparation of various nanofiber layers using wire electrospinning system. Arab. J. Chem..

[22] Mamidi N., Zuníga A.E., Villela-Castrejón J. (2020). Engineering and evaluation of forcespun functionalized carbon nano-onions reinforced poly (ε-caprolactone) composite nanofibers for pH-responsive drug release. Mater. Sci. Eng. C.

[23] Lu Y., Li Y., Zhang S., Xu G., Fu K., Lee H., Zhang X. (2013). Parameter study and characterization for polyacrylonitrile nanofibers fabricated via centrifugal spinning process. Eur. Polym. J..

[24] Chang W.M., Wang C.C., Chen C.Y. (2014). The combination of electrospinning and forcespinning: Effects on a viscoelastic jet and a single nanofiber. Chem. Eng. J..

[25] Lu B., Wang Y., Liu Y., Duan H., Zhou J., Zhang Z., Wang Y., Li X., Wang W., Lan W. (2010). Superhigh-throughput needleless electrospinning using a rotary cone as spinneret. Small.

[26] Dabirian F., Hosseini Ravandi S., Pishevar A. (2011). Investigation of Parameters Affecting PAN Nanofiber Production Using Electrical and Centrifugal Forces as a Novel Method. Curr. Nanosci..

[27] Müller F., Jokisch S., Bargel H., Scheibel T. (2020). Centrifugal Electrospinning Enables the Production of Meshes of Ultrathin Polymer Fibers. ACS Appl. Polym. Mater..

[28] Müller F., Zainuddin S., Scheibel T. (2020). Roll-to-Roll Production of Spider Silk Nanofiber Nonwoven Meshes Using Centrifugal Electrospinning for Filtration Applications. Molecules.

[29] Fong H., Chun I., Reneker D.H. (1999). Beaded nanofibers formed during electrospinning. Polymer.

[30] Weitz R.T., Harnau L., Rauschenbach S., Burghard M., Kern K. (2008). Polymer nanofibers via nozzle-free centrifugal spinning. Nano Lett..

[31] Gupta A., Harrison I.R. (1996). New Aspects in the Oxidative Stabilization of PAN-based Carbon Fibers: II. Carbon N. Y..

[32] Arshad S.N., Naraghi M., Chasiotis I. (2011). Strong carbon nanofibers from electrospun polyacrylonitrile. Carbon N. Y..

