# Supplementary material for: High Throughput Centrifugal Electrospinning of Polyacrylonitrile Nanofibers for Carbon Fiber Nonwovens
Source: Polymers (Basel). 2021 Apr 16;13(8):1313. doi: 10.3390/polym13081313 (PMC8072871; doi:10.3390/polym13081313)
Supplement: Supplementary file 1 [file polymers-13-01313-s001.zip › polymers-1167990-supplementary.pdf]

Supporting Information for:

High throughput Centrifugal Electrospinning of Polyacrylonitrile Nanofibers for Carbon Fiber Nonwovens

Andreas Hoffmann [1] and Alexander J. C. Kuehne [1, 2,*]

[1] Institute of Organic and Macromolecular Chemistry, Ulm University, Albert-Einstein-Allee 11, 89081 Ulm, Germany
[2] DWI—Leibniz Institute for Interactive Materials, Forckenbeckstraße 50, 52076 Aachen, Germany; * Correspondence: alexander.kuehne@uni-ulm.de

Experimental

PAN was obtained from Dralon GmbH, DMSO (99.9%) was obtained from Fischer Chemical and Acetone (technical grade) was obtained from Sigma Aldrich. The polymer solutions were prepared by dissolving the polymer at RT for 24 h, while stirring. The collector was a 35 µm thick aluminum foil, placed directly under a grounded iron plate. The samples were dried for 24 h at RT prior to analysis.

Rotational spinning of PAN-solutions in a binary DMSO:Acetone 8:2 mixture was conducted on an instrument from DIENES Apparatebau GmbH. The rotating bell was fed with the polymer solution with a constant rate of 9 mL/min.. The distance between bell and collector was held constant at 43 cm. A suction system, placed lateral and underneath the collector, was active during the spinning experiments, with a suction-power of 1000 m^2/h. The collector was connected to ground.

DSC measurements were performed with a heating rate of 10 K/min. with a N_2-flow of 50 mL/min..

TGA measurements were performed with a heating rate of 15 K/min. with a N_2-flow of 20 mL/min..

Parameter-variation in Fig. 1 a-d

The viscosity of the dope-solution was varied from m_{PAN} = 6 – 16 %, while keeping p = 0.75 bar, V = 60 kV, I = 150 µA and rh% > 30%. The rotation speed was varied from ω = 16 – 26 krpm to collect coherent nonwovens and avoid wet deposition, according to Figure 2. Higher rotation speeds for m_{PAN} = 14 and 16 % solutions explain the step in Figure 1.

The rotational speed was varied from ω = 10 – 30 krpm, while keeping, m_{PAN} = 14 %, p = 0.75 bar, V = 70 kV, I = 150 µA and rh% > 30%.

The voltage was varied from V = 40 – 70 kV, while keeping, m_{PAN} = 14 %, p = 0.75 bar, ω = 30 krpm, I = 150 µA and rh% > 30%.

The airflow was varied from p = 0.25 – 1.25 bar, while keeping, m_{PAN} = 14 %, V = 70 kV, ω = 30 krpm, I = 150 µA and rh% > 30%.

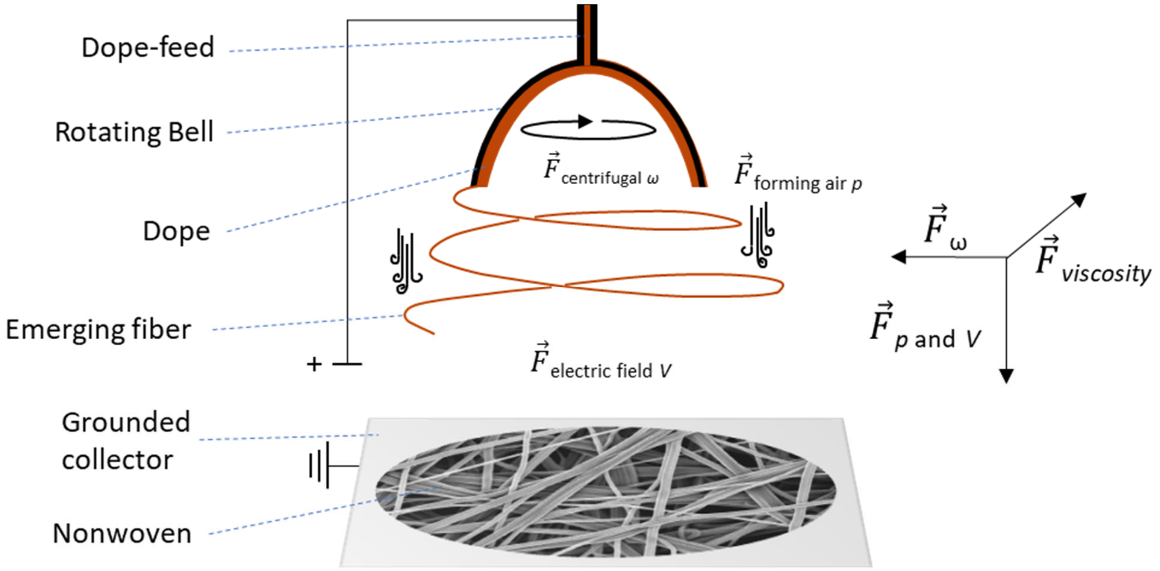

Figure S1: Scheme of the centrifugal electrospinning setup. The inner walls of a rotating bell are fed with a dope solution. An electric field, an air-stream and centrifugal forces apply on the polymer solution, leading to cannula-free formation of fibers from the edge of the bell. The emerging fibers are drawn towards a grounded collector underneath the bell. The direction of the individual drawing forces is indicated on the righthand side.

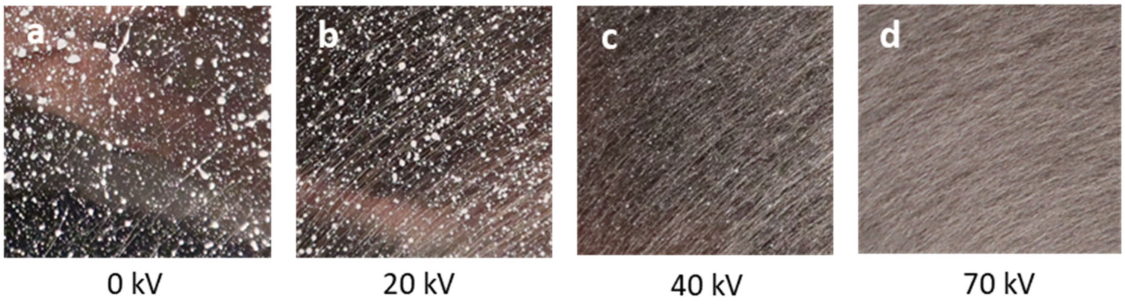

Figure S2: Photographs of the collector, after spinning at various voltages. The voltage was varied V = 0 kV (a) 20 kV (b) 40 kV (c) 70 kV (d), while keeping m_{PAN} = 14 %, p = 0.75 bar, ω = 30 krpm, I = 150 µA and rh% > 30% constant. Nonwoven with (almost) no droplets can be observed for Voltages > 40 kV.

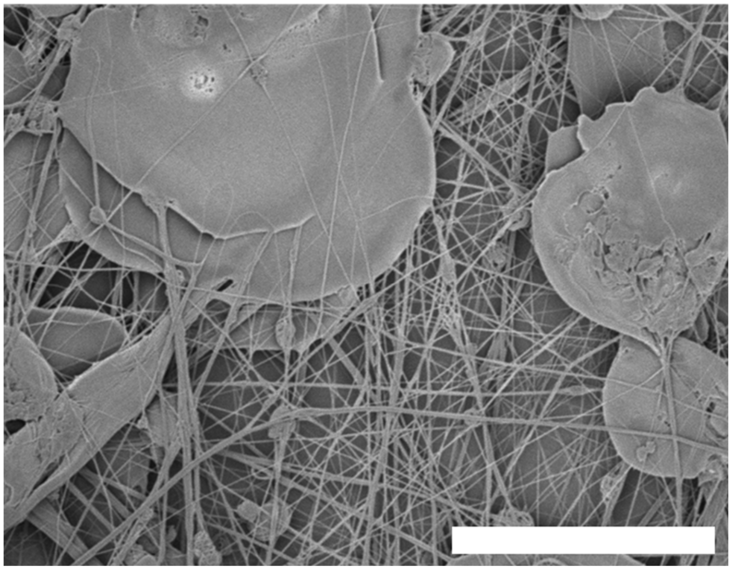

Figure S3: SEM-image of a nonwoven, spun from m_{PAN} = 6 %. Collection of Polymer-droplets on the nonwoven can be observed for $m_{PAN} \leq 6\%$. Spinning Parameters: p = 0.75 bar, V = 60 kV, I = 150 µA and $rh\%$ > 30%, ω = 16 krpm.

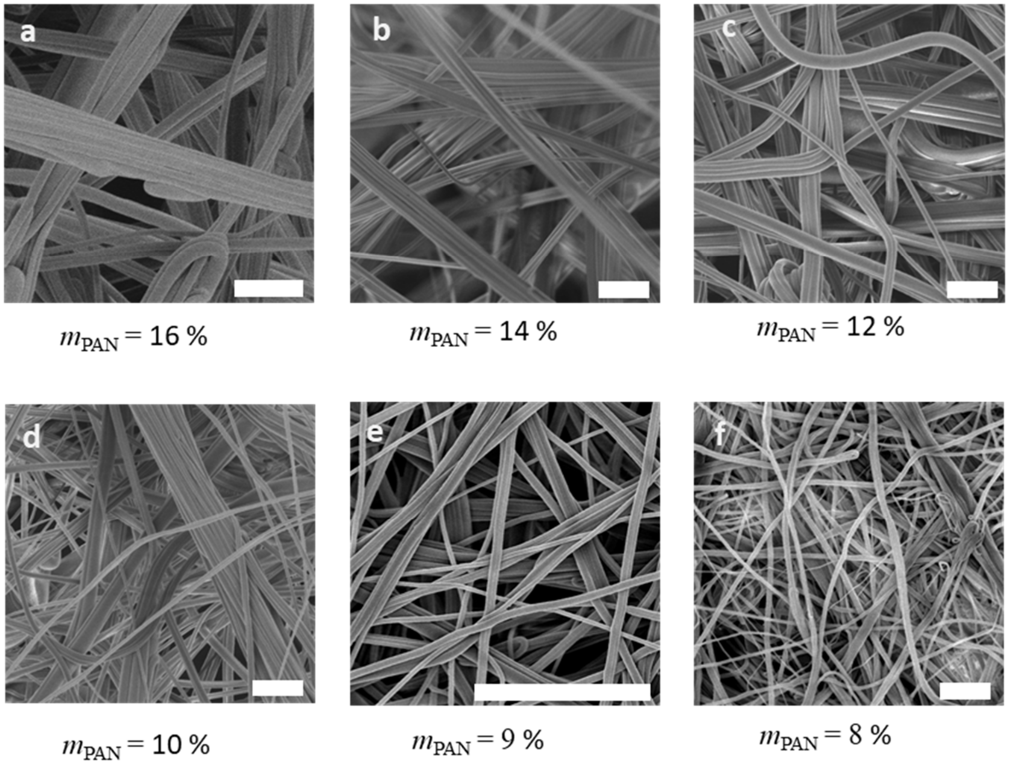

Figure S4: SEM-images of nonwoven, spun at various PAN concentrations. The viscosity of the dope-solution was varied m_{PAN} = 16 % (a), 14 % (b), 12 % (c), 10 % (d), 9 % (e), 8 (f) %, while keeping p = 0.75 bar, V = 70 kV, I = 150 µA and $rh\%$ > 30 % constant. The rotation speed was varied ω = 26 krpm (a), 26 krpm (b), 21 krpm (c), 16 krpm (d), 16 krpm (e), 16 krpm (f) to collect coherent nonwovens and avoid wet deposition, according to Figure 2. Emerging fibers with smaller diameters are less twisted. The scale bar represents 10 µm.

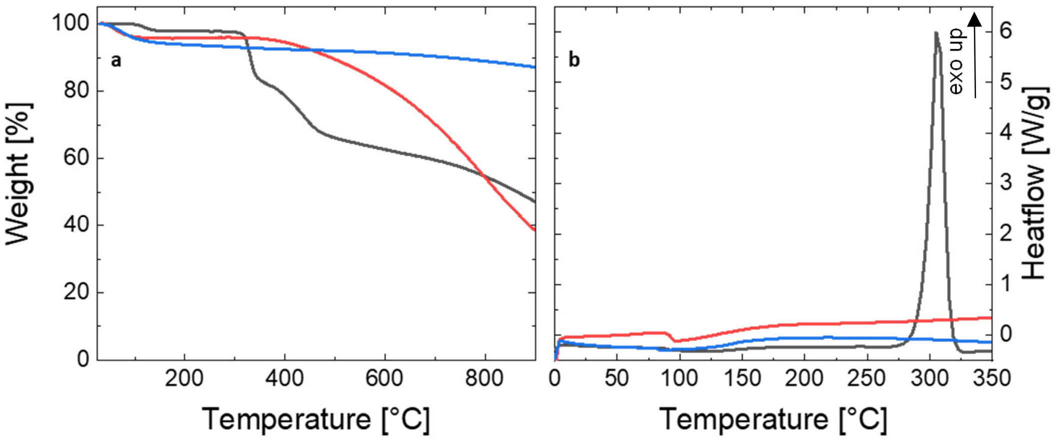

Figure S5: Thermal analysis of PAN-nonwoven. TGA- (left) and DSC-curves (right) of pristine (black), stabilized (red) and carbonized (blue) PAN-nonwoven. Both measurements were performed under N_2-atmosphere.

Analytic methods:

Scanning transmission electron microscopy (STEM): A Hitachi UHR-FESEM SU5200 is used for scanning transmission electron microscopy (STEM). The fibers were dried for 24 h and sputtered prior to analysis.
Fiber diameters were calculated by measuring at least 50 separate (untwisted) fibers.

Infrared-spectroscopy (IR): Infrared spectra were collected on a Perkin-Elmer Spectrum-two spectrometer with a diamond ATR-crystal.

Differential scanning calorimetry (DSC): DSC-measurements were performed on a Mettler-Toledo DSC 2 STARe System.

Thermogravimetric analysis (TGA): TGA- measurements were performed on a PerkinElmer TGA 8000.
